# Barriers and Facilitators to Career Progression Among International Nurses in Developed Countries: A Qualitative Meta‐Synthesis

**DOI:** 10.1155/jonm/2847893

**Published:** 2026-07-18

**Authors:** Ransford Akrong, Bibha Simkhada, Padam Simkhada, Precious Adade Duodu

**Affiliations:** ^1^ School of Human and Health Sciences, Department of Nursing, University of Huddersfield, Huddersfield, England, UK, hud.ac.uk; ^2^ Research and Innovation Office, University of Chester, Chester, England, UK, chester.ac.uk

## Abstract

**Aim:**

The systematic review aims to synthesise evidence on the barriers and facilitators of career progression of international nurses in developed countries.

**Background:**

International nurses migrate for career progression but face several challenges that impede their progress in their careers. However, evidence is scattered, and a comprehensive understanding is underdeveloped.

**Method:**

A systematic review and qualitative meta‐synthesis was carried out across PubMed, CINAHL, PsycINFO and Scopus. The review followed the PRISMA guidelines. Data were analysed using thematic synthesis and interpreted through the combined theoretical lenses of critical race theory and social justice theory to explore the structural and racialised dimensions of international nurses’ career progression. The Joanna Briggs critical appraisal tool for qualitative evidence was used to assess the methodological rigour of the papers.

**Results:**

Following critical appraisal, 15 studies were included that identified that communication and language, access to support and mentorship, discrimination and racism, and issues with social and cultural integration influence the career progression of international nurses.

**Discussion:**

Interpreted through critical race theory and social justice theory, the findings of this review point to a persistent lack of structural and cultural support for international nurses within host healthcare systems. Rather than isolated challenges, barriers such as communication difficulties, exclusion from training and limited mentorship reflect broader systemic issues, including institutional discrimination and unequal access to opportunity. These dynamics not only constrain international nurses’ career development but also risk undermining workforce diversity and retention.

**Conclusion:**

International nurses face systemic challenges to career progression; this highlights the urgent need for structural support, mentorship and equitable policies.

## 1. Background

A significant and growing deficit in the healthcare workforce presents a major challenge to health systems worldwide. This issue is attributed to various factors including chronic underinvestment in the healthcare sector, imbalances in workforce distribution, an ageing health worker population, retirement, burnout, high staff turnover, increased demand for healthcare services and poor working conditions [1]. This shortage is particularly concerning considering that the healthcare industry is one of the world’s largest employers. Health workers encompass a wide range of professionals, including doctors, nurses, aides, technicians and even medical waste handlers, who are all dedicated to delivering care to those in need. Despite having 6.5 times more health workers per capita than low‐ and middle‐income countries [2, 3], developed nations continue to experience a significant influx of international health workers, particularly nurses. Nurses and midwives represent nearly half (approximately 27 million) of the global health workforce of 65 million [2, 4]. The growing shortage of nurses is a pressing concern and can directly affect the quality of care provided in hospitals and other healthcare facilities.

The World Health Organization, in its most recent data, predicts that the shortage of nurses is expected to grow exponentially in the coming years with an additional 9 million nursing vacancies to be filled globally by 2030 [4]. The shortage has led to developed countries taking steps to fill in the gap, and one of these major steps have been to recruit nurses from other countries, mainly low‐middle‐income countries. Some of the steps include offering attractive incentives such as visa sponsorship programmes, accommodation and competitive salaries [5]. Countries such as the United Arab Emirates, the United Kingdom and Singapore have introduced various strategies to recruit international nurses, such as providing fair wages, free travel, language training and incentives for passing licensure examinations, easing the recognition of foreign professional qualifications and expediting visa approvals [6]. According to Article 4 of the WHO Global Code of Practice on the international recruitment of health personnel, it is recommended that destination countries implement measures to ensure equitable treatment of international and domestic nurses [7]. This includes providing them with equal access to educational opportunities and opportunities for career progression [4]. Developed countries which include the United States of America, United Kingdom, Australia, Germany and Canada are known to be the prominent destination countries that attract nurses from low‐middle‐income countries with approximately 15.2% of their total nurse workforce being from a foreign country [4].

International nurses are drawn to developed countries by the promise of a better life, access to higher education and opportunities for professional growth leading to higher salaries [8–10]. However, research suggests a significant disconnect between the expectations set for international nurses and their actual experiences. Garside et al. [11] found that international nurses often find themselves in lower‐level positions compared to the roles they held in their home countries, regardless of their previous experience and qualifications. Considering the motivations of international nurses for migrating overseas, career progression has stood out as one of the major reasons for relocating to the developed countries [9, 12]. Career progression is understood as advancement within nursing careers, including promotion, access to senior roles, leadership opportunities and movement into roles that reflect nurses’ competence and experience. While related concepts such as skill utilisation and workforce integration are inherently connected to career progression, they are conceptually distinct and are theorised accordingly throughout this review.

Additionally, reaching one’s career goals has been shown to be positively associated with job satisfaction among international nurses [13]. This is because professional satisfaction and role contentment are typically interconnected, and possibilities for growth are crucial [14]. International nurses, regardless of their experience level or country of origin, face significant obstacles in advancing their careers. A study of international nurses in Asia and the Middle East revealed that their career advancement was hindered despite meeting all qualifications [15]. A recent integrative review highlights how host countries have no procedures to enable international nurses to use their previously acquired skills into their new roles [16]. In the United Kingdom, it is found that international nurses’ career progression is much slower compared to their counterparts that are domestic nurses [9, 17].

Previous reviews have examined the general migration, transition and acculturation experiences of international nurses [18–24]. However, no review has focused specifically on career progression as a distinct workforce issue. To the best of our knowledge, this is the first systematic review to synthesise evidence on international nurses’ career progression through the lenses of critical race theory (CRT) and social justice theory. It therefore moves beyond describing transition challenges to showing how career progression is constrained by wider organisational and structural conditions within host healthcare systems.

### 1.1. Research Question

To achieve this study’s aim, the review seeks to answer the following question: What are the facilitators and barriers that impact the career progression of international nurses?

## 2. Method

The systematic review followed a step‐by‐step framework for developing a research question and inclusion criteria [25]. This systematic review was registered in PROSPERO with the registration number CRD42024567663. The study relied on peer‐reviewed articles available in reputable databases, including PubMed, CINAHL, Scopus and PsychINFO, published from January 2000 to January 2025. The timeframe was to capture contemporary evidence on international nurses within the context of increased globalisation and international recruitment in high‐income countries. Keywords were utilised, considering synonyms, related phrases and variations. The search technique was customised for each database, using Boolean operators to ensure efficient retrieval. Keywords used for this study include career progression, international nurses, support, discrimination and opportunities. In addition to database searching, the reference lists of all included studies were manually screened to identify additional eligible studies that may not have been captured through the database search. The complete search strategy for each database, including Boolean operators and search terms, is presented in Appendix A. As stated in Table 1 below, each of the selected studies included in this systematic review had to meet certain inclusion requirements.

**TABLE 1:** Eligibility criteria.

	Inclusion Criteria	Exclusion criteria
Sample (S)	Internationally recruited nurses (nurses educated and registered in one country before being recruited to work in another) travelling abroad to work in any health or care setting	Allied health professionals Student health professionals Domestically trained nurses Any other profession
Phenomenon of interest (P of I)	Barriers and facilitators of international nurses’ career progression.	Studies discussing any other phenomenon such as integration, transition, recruitment and migration.
Design (D)	Interview, focus group, case study or observational study	Nonqualitative approaches
Evaluation (E)	The perceptions, attitudes, views and feelings of international nurses working in a country different to the one they were initially educated and registered in.	Studies published before the year 2000 to ensure relevance to contemporary nursing practice and career dynamics
Research type (R)	Qualitative primary research	Quantitative primary research Secondary research

## 3. Data Extraction

This study followed the Preferred Reporting Items for Systematic Reviews and Meta‐Analyses (PRISMA) checklist [26]. Employing a reporting checklist is crucial for evaluating the quality of all retrieved papers and improving the reporting of this review [27]. Two reviewers independently screened titles, abstracts and full texts against the eligibility criteria. Disagreements were first resolved through discussion between the two reviewers. Where consensus was not reached, a third reviewer acted as an arbitrator. Studies that did not fulfil the eligibility criteria were excluded. The articles selected to be included in the review were extracted in a spreadsheet using Microsoft Excel. The author list, publication year, study setting, study design, population characteristics, methodology and key relevant findings of the study were extracted. The screening phases were depicted using the PRISMA flow diagram in the final report. Table 2 below shows a summary of data extracted from the included studies.

**TABLE 2:** Summary of data extraction.

SN	Author, year	Country	Study aims	Method and data collection and analysis	Summary of relevant findings to the current study
1	Kamau et al., [33].	Finland	To describe culturally and linguistically diverse registered nurses’ experiences of integration	Qualitative descriptive design Semistructured interviews Content analysis	Poor language sufficiency Lack of language and communication support Discrimination
2	Estacio and Saidy‐Khan, [36].	United Kingdom	To explore the experiences of racial microaggression among migrant nurses. Thematic analysis	Qualitative Reflective diary writing	Verbal abuse Discrimination Unbalanced allocation of training courses Denial of opportunities to develop their knowledge and skills
3	Henry, [34].	United Kingdom	Explore the perceptions of career progression in the NHS of a group of midwives and nurses trained in Ghana and working in the United Kingdom	Qualitative Semistructured interviews; Thematic analysis	Inadequate language support Discrimination and racism Lack of official support from managers Unfamiliarity with the UK promotion process
4	Wheeler and Foster [42].	USA	To explore nurses’ perspectives and experiences regarding their participation in hospital governance structures and their plans for professional advancement to understand the value they place on the process	Explorative qualitative study Semistructured interviews Constant comparative method	Lack of time and cost of participating in governance Lack of desire to leave the bedside Unwillingness to take up extra responsibility Lack of guidance and encouragement from supervisors Discrimination regarding level of support provided
5	Iheduru‐Anderson, [32].	USA	To describe Black African‐born nurses’ perceptions of their nonnative accent’s effect on their career advancement to management or faculty roles	Qualitative research focused on ethnographic design Individual interviews Thematic analysis	Language and communication challenges Discrimination and racism Lack of organisational support Stereotypes about Black culture
6	O’Brien and Ackroyd, [40]	United Kingdom	To examine the factors that impact overseas nurses’ assimilation	Comparative case study Qualitative study Observations, semistructured interviews Thematic analysis	Exclusion from skilled tasks despite qualifications Mentors felt burdened and frustrated Lack of time/effort to properly support nurses
7	Salma, [35].	Canada	To look at the perceptions of internationally educated nurses regarding career advancement and educational opportunities in	Interpretive descriptive study semistructured interviews Thematic analysis	Poor communication blocked access to education Discrimination and racism Lack of support and mentorship Social barriers
8	Likupe, [37].	United Kingdom	To explore experiences of racism, discrimination and equality of opportunity among Black African nurses and their managers’ perspective	Qualitative FGD and semistructured interviews Thematic analysis	Lack of skills recognition Unequal allocation training No guidance or career courses Lack of encouragement from managers
9	Ugiagbe, [39]	United Kingdom	To explore participants’ perspectives on integration and how they attained, sustained and thrived in senior clinical and management positions	Qualitative Semistructured interviews Interpretive phenomenological analysis (IPA)	Unequal access to career advancement Equal opportunity policies poorly implemented Lack of transparency in the promotion process Lack of support systems for development
10	Alexis, Vydelingum, and Robbins, [38].	United Kingdom	To explore the experiences of overseas Black and minority ethnic nurses employed in an acute in National Health Service (NHS) hospital in the south of England	Qualitative Semistructured in‐depth interviews Thematic analysis	Lack of equal opportunities Racism and discrimination
11	Allen, [31]	USA	To explore the experiences of internationally educated nurses in management positions in the United States healthcare organisations to understand the obstacles and support these individuals’ experience when pursuing and working in managerial roles	Qualitative phenomenological study Semistructured interviews Colaizzi’s seven‐step phenomenological process	Language and communication challenges Cultural differences impacted social interaction and work experience Supervisors encouraged nurses to take leadership roles Support from staff and supervisors helped achieve success Opportunities for growth and further training
12	Larsen, [41].	United Kingdom	To examine how discrimination affects the career progression of overseas nurses	Existential phenomenological analysis	Experienced blatant racism Discrimination affected self‐worth
13	Belita and Ford, [44]	Canada	To explore these challenges and describe the educational program which adopts a tailored mentoring approach to facilitate their successful completion of the registration examination	Two in‐depth interviews with two overseas nurses Participatory action research Individual interviews Thematic content analysis	Resilience helped cope with discrimination Internalised stigma Lack of support to understand new norms Mentorship helped with success Learnt and adapted to new nursing culture
14	Adhikari and Melia, [14]	United Kingdom	To examine Nepali migrant nurses’ professional life in the United Kingdom	Multisited ethnographic approach In‐depth interviews Thematic analysis	Discrimination with role assigning No support for career progression
15	Daniel et al., [43]	United Kingdom	To identify initial expectations and experiences on Filipino nurses	Qualitative method FGDs Thematic analysis	Unequal access to career advancement Culturally sensitive orientation was helpful Support helped reduce stress Adapting to the UK health system was stressful

### 3.1. Risk of Bias (Quality) Assessment

This systematic review holds promise for informing policies in the recruitment and retention as well as the professional development of international nurses. The methodological quality of the included studies was assessed independently by two reviewers. The methodological quality of the included studies was critically appraised using the Joanna Briggs Institute standardised critical appraisal checklist for qualitative studies to ensure rigour and reliability in the assessment of evidence [28]. This helped to determine the validity and trustworthiness of the research findings and inform decision‐making processes based on the best available evidence. The methodological quality of each study was rated as low (0%–60%), medium (61%–79%) or high (80%–100%). Of the 15 included studies, 11 studies were rated as high quality, three studies as medium quality and one study as low quality, with common methodological limitations including inadequate reporting of the researcher’s cultural or theoretical positioning and limited discussion of the influence of the researcher on the research process. In instances of disagreement, a third reviewer was consulted to reach a consensus.

As this review synthesised previously published studies, ethical approval was not required for the present review. However, ethical reporting within the included studies was considered during quality appraisal. The included studies were assessed for whether they reported ethical approval, informed consent, participant confidentiality and appropriate protection of participants. Where ethical approval was not clearly stated, this was noted as a limitation in the appraisal process.

### 3.2. Analysis and Synthesis

After data extraction, thematic analysis [29] was conducted to explore key patterns within the papers to compare the findings across the selected articles. The data extracted from the selected papers were read and reread to familiarise with the data, and initial codes were generated. The next stage was to search for themes from the codes by grouping codes that portrayed similar ideas. The themes were reviewed, defined and named afterward. Two reviewers independently coded the findings from the extracted data to develop themes. To ensure consistency in the coding process, inter‐rater reliability was assessed by comparing initial codes across coders to establish the degree of agreement in their decisions [30].

### 3.3. Study Inclusion

**FIGURE 1:**
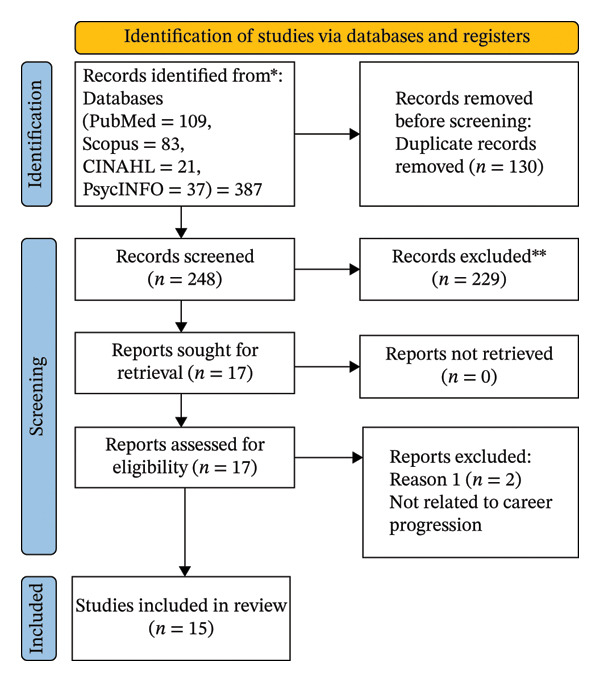
PRISMA flow diagram.

A total of 376 studies were retrieved from the search. A total of 130 duplicates were removed, and 246 titles and abstracts were screened. Seventeen studies were screened for full text, and 15 studies were selected based on the inclusion and exclusion criteria outlined for the review. Although the final synthesis included 15 studies, this was considered sufficient for a qualitative meta‐synthesis because the review focused specifically on career progression among international nurses rather than their general transition experiences. The included studies provided rich qualitative data across four developed‐country contexts. Therefore, the value of the sample lies in the depth and relevance of the evidence rather than the number of included studies alone. Additionally, this review examines developed countries as primary destinations for international nurses, highlighting their promotion, and workforce development systems. These countries depend significantly on international recruitment to mitigate nursing shortages, providing a relevant context for analysing career progression postmigration. The PRISMA flow diagram [26] (Figure 1) was completed to record each step of the search process and the number of included studies at each stage.

### 3.4. Findings of the Review

The research included studies from Finland (1), the United Kingdom (9), the USA (3) and Canada (2). Synthesis of the review is organised into four main themes which are associated to the career progression of international nurses: language and communication; discrimination and racism; support and mentorship; and cultural and social integration.

### 3.5. Language and Communication

Language and communication play a significant role in the career progression of international nurses. The findings revealed that international nurses face challenges such as limited access to opportunities and limited career development because of their poor language skills [31–33]. For example, an international nurse explains why her White colleague gains a promotion in her place by mentioning that ‘*she got it (promotion) because the questions they asked and everything, it’s her natural language, that’s her natural expression … for the African you have to prod … because that is the way we do things, you know. So the panel who are not used to the Africans, their way of life, or mannerisms, they say that we are thick, we cannot perform*’ [34]. Accent and communication differences affected how competence was judged, particularly in relation to leadership and promotion opportunities [32, 35]. In Finland, international nurses personally believed that their inabilities to communicate fluently in the Finnish language may hinder their abilities to progress in their carrier. The quote below illustrates how an international nurse felt about her communication challenges.

“I don′t think I have plans to continue being a nurse in this country because of these challenges because I don′t feel challenged enough as a nurse because I feel like I have really strong capabilities, I am able to really do much, but the language will always limit me” (Participant 012; [31]; page 3)

Other challenges international nurses face regarding communication and language barriers are their difficulty to understand nonverbal communication and slangs used in the clinical setting [35]. Additionally, poor communication and difficulty in communication hindered the ability of international nurses to pursue further education and training needs that would support their career progression [31, 35]. International nurses struggled to build strong social networks with domestic nurses, and this affected their career progression because of the potential lack of support and guidance [35].

### 3.6. Discrimination and Racism

International nurses are faced with several layers of stigma and discrimination that affect their tendency to progress in their career. These forms of discrimination play out in various ways which include racial microaggressions [35–37]. International nurses are often verbally abused by their superiors, who are mostly domestic nurses from several countries, aiming to demean international nurses and make them feel incompetent [36]. There were instances where international nurses were denied opportunities to progress in their career as leadership positions were only assigned to domestic nurses [33, 36–38]. For some, junior domestic nurses were promoted ahead of international nurses who possessed more working experience and higher qualifications [37]. For instance, a participant relayed that ‘*I have seen final year students, mentor them and only to see them being my seniors*’ [36]. Additionally, international nurses encountered discrimination during the processes of promotion in their respective organisations [34]. This is manifested in the gaps of knowledge regarding a comprehensive understanding of the promotion processes in their organisations [34, 37].

#### 3.6.1. Training Allocations

International nurses encounter unbalanced training allocation when they seek to pursue training opportunities that will potentially impact their progression [34, 36–38]. Training opportunities are mostly assigned to domestic nurses with only a few international nurses being allocated to these trainings. For example, a nurse relayed that ‘*I have also questioned the criteria of sending nurses for different courses within the Trust. I always put my name first on the list but to my amazement I have never been listed for interviews*… *I feel when I go back home I will be still the same nurse I came with the same qualifications. I expected that when I go back home I will be holding degrees and different courses of achievement*’ [36]. In another instance, an international nurse mentioned that ‘*There are times when I have asked to go for a course, like me and another person want to go for the same course, they will choose their own people*’ [37]. In situations where international nurses are considered for these trainings, they find that most of these trainings are irrelevant to their current job description and career progression [34].

Some international nurses felt they lacked the needed support from their managers to pursue further training. This lack of support manifested in various forms including denail of study leave, absolute denial to pursue courses, inconsiderate training time allocations, lack of funding, and discouragement from pursuing training opportunities [33–36, 38, 39].

#### 3.6.2. Task Allocation

International nurses also experienced challenges with being excluded from higher status tasks that could help with their professional development to progress in their career [14, 33, 40]. International nurses were mostly subjected to only direct care tasks and excluded from team‐based tasks to help them build their teamwork skills [40]. Also, international nurses felt there were internal closures in the organisations where they were automatically sidelined and not given access to higher‐skilled tasks [40].

#### 3.6.3. Issues of Stigma

The level of stigma encountered by international nurses took the form of perceived inferiority by their domestic managers and colleagues because of their culture, race and ethnicity which led to their capabilities being questioned and undermined [32, 40]. These acts of stigma and discrimination are often internalised by international nurses, which leads to lack of confidence and tendency to seek career progression opportunities [41]. Some international nurses relayed that

*“When the Indian nurses were recruited there was this thing (policy) about recruitment and retention so you find that the kind of treatment that they were given is that kind of treatment that would want to keep people where they are and then after a while it (the feeling that Indian nurses were recruited to be retained) all worn off and that’s why they came up with this attitude that they are better than you because they were given the impression that they were needed more than you are”.* [37].

*“Nobody recognises any black (nurse) no matter how intelligent you are. If you are intelligent they would rather prove you to be too confrontational. So I tell you I cannot hide, I told my manager last week I said I’m not happy”* [37].

While there were cases of racism and discrimination, some individuals managed to be resilient and cope with instances of racism to progress in their careers regardless [41]. Other strategies also involved resisting racism through meaning making (Larsen 2007).

## 4. Support and Mentorship

International nurses lacked the needed support to progress in their careers in their respective host countries. In some cases, international nurses perceived that the level of support they received from their managers was only a matter of pretence, as they thought that managers only encouraged them to go for training just to appear to be providing support for them [34]. Support was experienced as unevenly distributed, particularly in relation to interview preparation, mentoring and access to guidance [34, 40, 42]. According to one nurse,

“*you hear the manager or some senior member of staff call a colleague who is also going for the interview, they go to office for a long time and they come back they are doing this (and) that … (but) nobody even asks me … have you prepared or is somebody helping you prepare for the interview? No-one, no senior member of staff ever called me or asked me*” [34].

Evidence shows that managers perceived that mentoring international nurses was very demanding and often frustrating [40]. International nurses lacked the motivation to pursue career progression because they did not receive any form of guidance and encouragement from their supervisors to do so [32, 42]. Other studies found that international nurses did not know that they were entitled to any support in their organisations [34].

There was the challenge of lack of skills recognition among international nurses, which impeded the level of support that they could have got to progress in their respective careers in their host countries [32, 35, 37, 38, 40, 41]. In one study, managers relayed that they simply did not have the time to explore the possibility that international nurses were qualified to perform tasks assigned to them [40]. Another challenge was a lack of a robust grievance system as international nurses felt their problems were not addressed by their managers [34].

International nurses experienced a lack of support regarding the promotion processes in their host organisations. Support in preparing for interviews was absent for international nurses, and in some cases, they found it inadequate [34]. One study, however, identified that some international nurses have received support through coaching from their superiors, and this led to their career progression [39].

## 5. Cultural and Social Integration

International nurses’ career progression is affected by social and cultural integration. Many international nurses struggle with adjusting to new healthcare systems and cultural expectations, which can impede their professional growth. For instance, Adhikari and Melia [14] highlighted that Nepali international nurses, despite being highly qualified and experienced in specialised areas such as critical care and management, often end up working in long‐term care sectors providing personal care for the elderly. This mismatch between their qualifications and job roles leads to frustration and a lack of job satisfaction. Additionally, Daniel et al. [43] noted that newly recruited Filipino nurses experienced stress while adjusting to the new system of healthcare in the United Kingdom. Some international nurses were reported to be unfamiliar with the promotion process in their host countries, impeding their ability to properly prepare themselves to progress in their respective careers [34]. However, these challenges were mitigated through the provision of culturally sensitive programmes [44]. Social integration was important because it shaped access to workplace relationships, informal knowledge and confidence in navigating career pathways. Overall, fostering an inclusive environment that acknowledges and addresses cultural differences is essential for the career advancement of international nurses.

“*My mentors were always available when I needed them and took time necessary on an individual level when needed and always find time to follow up or call us at home regarding our progress.”* [44].

Other social issues that affected the career progression of international nurses include individual and family responsibilities. International nurses with families and other dependents felt constrained to take up opportunities that would potentially increase their chances that would enable them progress. For instance, an international nurse would have to work full time to progress in their career but can only work for restricted hours because of their dependents. An example of such instance is shown in the quote below.

*“I started working part-time because when I came here I didn’t know anybody. I had two kids who were going to school. I didn’t have babysitters”* [35].

As a result, some international nurses prioritised caring for their family members instead of using that time to pursue further educational credentialing or working full time and committing to a fixed working schedule [35]. On the individual level, some international nurses perceived that pursuing higher roles led to more responsibilities than they can handle [42]. Some nurses also mentioned that the additional responsibilities that come with progressing in your career led to stress and discomfort [42].

## 6. Discussion

This review has explored the indicators of career progression of international nurses who have migrated to work in developed countries, predominantly from low‐ and middle‐income countries. The findings reveal an interplay of factors that affect the career progression for international nurses. These factors include communication and language barriers, discrimination and racism, lack of mentorship and support and issues regarding cultural and social integration. This review interprets these findings through the combined theoretical lenses of CRT and social justice theory to explain the structural conditions that shape international nurses’ career trajectories. CRT posits that racism is not an aberration within social and institutional systems but is embedded structurally within their policies, cultures and everyday practices, producing racially unequal outcomes even in the absence of explicitly racist intent [45]. Social justice theory complements this by arguing that the unequal distribution of opportunities, resources and recognition across groups constitutes an ethical failure that institutions have a positive obligation to address [46]. Applied together, these frameworks reveal that the barriers identified in this review are not isolated challenges but are interconnected expressions of structural racism and institutional injustice that systematically constrain the career progression of international nurses within host healthcare systems. Applying these frameworks, the evidence suggests that systemic change is required to mitigate inequities and enable equitable career progression.

A key interpretive insight emerging from this synthesis is that barriers to career progression may not operate independently but appear to be mutually reinforcing, suggesting a compounding pattern of disadvantage that could be more restrictive in combination than any single barrier alone. Language and communication challenges reduce confidence and social capital, limiting access to informal professional networks, reducing opportunities for mentorship, restricting access to training and promotion and ultimately perpetuating workforce stratification that concentrates international nurses in lower‐status roles regardless of their qualifications and experience. Discrimination and racism operate as accelerants at every stage of this chain, amplifying the effect of each barrier and narrowing the pathways through which individual resilience or institutional goodwill might otherwise enable progression. This compounding dynamic has not been sufficiently articulated in prior reviews of international nurses’ experiences, which have tended to treat barriers categorically rather than as structurally interrelated mechanisms of exclusion.

Language and communication barriers were identified across the literature as significant constraints on IENs’ career progression, limiting access to training, mentorship and leadership opportunities. The stigmatisation of accents, the perception that nonnative speakers are less competent and the exclusion of IENs from leadership roles on the basis of communication style all reflect what CRT scholars identify as epistemic bias, implying the systematic privileging of knowledge, expertise and modes of expression that originate within the dominant cultural and linguistic norms of the host country and the corresponding devaluation of those that originate elsewhere [45]. The perception that international nurses are incompetent due to their communication differences further demonstrates how epistemic bias functions as a gatekeeping mechanism within healthcare systems, restricting access to roles that reflect nurses’ actual capabilities. This aligns with prior research suggesting that language barriers limit access to training and mentorship necessary for career progression [47, 48]. From a social justice theory perspective, this reflects a failure of procedural justice, as the competence assessment processes and opportunity allocations are biased against IENs. Healthcare organisations have an ethical responsibility to rectify this by implementing culturally inclusive assessment frameworks and providing targeted communication support [49]. These findings suggest that interventions targeting language and communication support could enhance career progression opportunities for international nurses.

The limited mentorship and support available to international nurses can be understood through CRT, particularly the concept of interest convergence, which suggests that the interests of minoritised groups are supported only when they align with those of dominant groups [46]. The findings indicate that while international nurses are actively recruited to address workforce shortages, they are not consistently supported with the mentorship, training access or guidance needed for career progression, suggesting that their recruitment serves organisational needs, but their advancement is not equally prioritised. Evidence from the included studies reinforces this pattern, with managers sometimes perceiving mentoring international nurses as more demanding [40], training opportunities more often allocated to domestically trained nurses [17] and limited support for promotion preparation [34]. Notably, where mentorship and coaching were provided, the findings suggest meaningful benefits for career progression. Ugiagbe [39] found that international nurses who received coaching and guidance from senior colleagues were able to progress in their careers, illustrating that structured mentorship can directly counteract the gatekeeping mechanisms discussed above when organisations actively invest in it. From a social justice theory perspective, this represents what genuine distributive justice can look like in practice. However, this finding stands in contrast to the broader pattern identified across the included studies, where access to such support remained uneven and inconsistently available, particularly across racial and national lines.

Discrimination and racism represent the most visible expression of the structural dynamics highlighted by CRT. The evidence of racial microaggressions, exclusion from leadership and managerial roles, unequal disciplinary processes and the promotion of less experienced domestic nurses over more experienced international nurses reflects the persistence of racism within institutional structures [46]. These patterns show how unequal outcomes are reproduced through everyday organisational practices, informal norms and managerial decisions that may appear neutral to those not affected. Participants’ perceptions that some institutions were inherently racist, alongside the selective enforcement of equality policies, further suggest that in the contexts described by participants, formal commitments to diversity may coexist with racial inequality where underlying organisational structures are not actively addressed. These findings may also reflect a pattern of workforce stratification, whereby international nurses described being concentrated in lower‐status roles that did not appear to reflect their qualifications or expertise, potentially reinforcing existing hierarchies within the healthcare systems represented in this review [16, 50]. Despite these pervasive structural barriers, some international nurses demonstrated resilience and actively resisted the effects of racism through processes of meaning‐making, enabling them to continue progressing in their careers despite discriminatory conditions [41]. Interpreted through CRT’s concept of counter‐storytelling, these accounts of resilience can be understood not as evidence that existing structures are adequate but as evidence of individual agency operating in spite of, rather than because of, institutional support. The experiential accounts of international nurses are particularly important in revealing these patterns, as they highlight forms of structural inequality that are often not captured in formal policies or workforce data. From a social justice perspective, this represents a serious ethical concern, as the unequal distribution of opportunities, inconsistent application of organisational processes and normalisation of discriminatory practices reflect failures in both fairness and accountability that require structural change.

Challenges in cultural and social integration further show that career progression is shaped by structural conditions rather than individual adaptation alone. The findings indicate that limited social integration restricted international nurses’ access to informal networks, institutional knowledge and the relationships through which career opportunities are often shared and accessed. This reflects the argument within CRT that exclusion does not occur only through overt discrimination but also through everyday workplace structures that privilege those already familiar with dominant cultural norms. When international nurses are excluded from informal conversations, collegial relationships and social networks, they are not only socially isolated but also limited in their ability to access opportunities for progression. Evidence that culturally sensitive orientation programmes helped reduce some of these challenges [44] suggests that targeted interventions can improve integration. Participants in this study described mentors who were consistently available, took time to understand their individual needs and followed up on their progress beyond formal expectations, illustrating how sustained and personalised support can foster a genuine sense of belonging and confidence among international nurses. From a social justice theory perspective, this reflects what recognition justice looks like when institutions actively acknowledge and accommodate the cultural identities and adjustment needs of international nurses, rather than expecting integration to occur through individual adaptation alone. However, their absence in many settings in this review means that cultural marginalisation continues to act as a barrier to advancement.

The review highlights that barriers to career progression for international nurses are deeply rooted in structural racial inequality and institutional injustice, manifesting across various countries and healthcare systems. Using CRT and social justice theory, it reveals an interconnected system of language marginalisation, racial exclusion, institutional gatekeeping and cultural isolation that together hinder professional advancement for international nurses despite their qualifications. The findings of this review suggest that the healthcare systems represented in the included studies may not have been designed with the integration and advancement of international nurses in mind and that sustained institutional commitment to addressing these inequalities appears, at present, to be limited. The findings also suggest that significant ethical implications, as discriminatory practices, limited mentorship and nonrecognition of qualifications violate principles of fairness and equal opportunity. Healthcare organisations have both a strategic interest and ethical duty to create transparent and equitable career progression systems that actively combat racialized disadvantages through dedicated policy and leadership efforts.

### 6.1. Limitations

The review’s limitation should be considered within the scope of the study. While nurses migrate to several countries to work, the review explored experiences of international nurses in developed countries only. Additionally, since this review focuses solely on publications that employed qualitative papers only, it may lack perspectives and information provided by quantitative studies on the topic. Furthermore, there is a potential for publication bias from English‐language–only studies. The review excluded grey literature which could have provided further information and helped contextualise the issues regarding nurses’ career progression. A limitation of the review is that ethical reporting varied across the included studies, and not all studies provided detailed information on ethical approval or consent procedures. Lastly, the included studies were conducted across different countries, including the United Kingdom, the United States, Canada and Finland, each with distinct healthcare systems, regulatory frameworks and workforce policies. These contextual differences may influence how barriers such as language requirements, discrimination and access to career progression opportunities are experienced. While common patterns were identified across studies, some findings may be shaped by country‐specific systems and should therefore be interpreted with consideration of contextual variation.

## 7. Conclusion

Our research demonstrates that international nurses are faced with a complexity of challenges that impede their career progression in their host countries with little to no support observed. There is the need for a multifaceted approach to address these challenges. Policymakers and recruitment agencies of international nurses should design initiatives to improve existing language and communication support provided to international nurses. These language support programmes should include clinical communication nuances and implement guidelines to encourage inclusive communication practices to reduce discrimination based on accents. Furthermore, while enhancing organisational support provided to international nurses, it is important to find innovative means to combat discrimination and racism that international nurses face and promote cultural and social integration. We recommend that promotion processes in healthcare sector be monitored to ensure fairness and promote equal opportunity for international nurses. Additionally, employers should monitor and ensure that there is equitable access to training and educational opportunities for international nurses. Mentorship schemes in healthcare organisations should be improved by equipping mentors with the right tools and guidance on how to support international nurses. Further research should be conducted to explore managers’ perception on international nurses’ career progression experiences and to understand the support available to international nurses regarding their career progression. Additionally, a longitudinal study is needed to track the career trajectories of international nurses examining the long‐term impacts of language and support initiatives, mentorship programmes and antiracism and discriminatory trainings.

### 7.1. Implications for Nursing and Health Policy

The systematic review shows the need for nursing leaders and healthcare organisations that recruit international nurses to prioritise their career progression in their workforce retention and inclusive strategies. Key implications include the implementation of strategies such as continuous clinical communication support, transparent promotion processes, mentorship programmes and culturally sensitive initiatives. Leaders of various health organisations should make continuous effort to address systemic issues such as discrimination and racism by ensuring equal access to trainings. National policies should focus more on long‐term career sustainability and move beyond recruitment. Strengthening support for IENs will not only improve retention and job satisfaction but also enhance equity, cultural competence and workforce stability within diverse healthcare systems.

The findings also have implications for global workforce equity. When developed countries recruit international nurses from low‐ and middle‐income countries but fail to recognise their skills, support their progression or provide equitable career opportunities, this creates a double inequity. Source countries lose skilled professionals, while destination countries may underuse their expertise. Ethical international recruitment therefore requires more than filling workforce gaps; it must include fair integration, skill recognition and career development for international nurses.

## Author Contributions

Study design: Ransford Akrong, Bibha Simkhada and Padam Simkhada.

Data collection and data analysis: Ransford Akrong, Bibha Simkhada, Padam Simkhada and Precious Adade Duodu.

Study supervision and critical revisions for important intellectual content: Bibha Simkhada and Padam Simkhada.

Manuscript writing: Ransford Akrong, Bibha Simkhada, Padam Simkhada and Precious Adade Duodu.

## Funding

The study was funded by the University of Huddersfield as part of the first author’s PhD scholarship.

## Ethics Statement

Ethical approval was not required because the review drew upon literature within the public domain.

## Conflicts of Interest

The authors declare no conflicts of interest.

## Data Availability

The data extracted and synthesised in this review are derived from previously published studies, all of which are cited within the manuscript and listed in the reference list. The data extraction spreadsheet and coding framework generated during this review are available from the corresponding author upon reasonable request.
